# Identification of *Striga hermonthica*-Resistant Upland Rice Varieties in Sudan and Their Resistance Phenotypes

**DOI:** 10.3389/fpls.2016.00634

**Published:** 2016-05-13

**Authors:** Hiroaki Samejima, Abdel G. Babiker, Ahmed Mustafa, Yukihiro Sugimoto

**Affiliations:** ^1^Graduate School of Agricultural Science, Kobe UniversityKobe, Japan; ^2^International Cooperation Center for Agricultural Education, Nagoya UniversityNagoya, Japan; ^3^College of Agricultural Studies, Sudan University of Science and TechnologyKhartoum North, Sudan; ^4^Gezira Research Station, Agricultural Research CorporationWad Madani, Sudan

**Keywords:** NERICA, parasitic weed, pre-attachment, post-attachment, rhizotron

## Abstract

Rice has become a major staple cereal in sub-Saharan Africa. Currently, upland rice cultivation is expanding particularly in rainfed areas where the root parasitic weed *Striga hermonthica*, a major constraint to cereal production, is endemic. Laboratory, pot, and semi-controlled open air experiments were performed to evaluate resistance of selected rice varieties in Sudan to a resident *S. hermonthica* population. In the laboratory, 27 varieties were screened for post-attachment resistance using the rhizotron technique. Varieties displaying high post-attachment resistance, Umgar, NERICA5, and NERICA13 together with NERICA4, NERICA18, and Nipponbare, a lowland rice variety, were further evaluated for performance and *Striga* resistance in pot and semi-controlled open air experiments and for germination inducing activity in a laboratory. In addition, comparative studies on reaction of Umgar, Kosti1 and Kosti2, released varieties for commercial production in Sudan, to the parasite were performed in two pot experiments. In the pot experiments Umgar and NERICA5, consistently, sustained the lowest *Striga* emergence (<2.2 *Striga* plants per pot), while NERICA13 and NERICA4 supported 1.8–5.7 and 8.7–16.4 *Striga* plants per pot, respectively. In an artificially *Striga*-infested field, number of emergent *Striga* plants per 10 rice hills, at harvest, was 2.0, 2.0, 4.8, 13.5, 13.3, and 18.3 on Umgar, NERICA5, NERICA13, NERICA4, NERICA18, and Nipponbare, respectively. *Striga* had no adverse effects on total above-ground parts and panicle dry weight in Umgar and NERICA5. Germination-inducing activity of root exudates, at 14 days after sowing onward, was markedly lower for Umgar than for NERICA5, NERICA13, NERICA4, and NERICA18. Based on these findings, Umgar has both pre and post-attachment resistance to a resident *Striga* population in Sudan. Kosti1 and Kosti2 did not exhibit *Striga*-resistance at the same level as Umgar. Further the resistance of NERICA5, a variety reported to be endowed with a broad spectrum resistance to *Striga* species and ecotypes, at least to one resident *Striga* population in Sudan was clearly indicated.

## Introduction

Rice is becoming an important staple crop in Africa. From 1984 to 2013 the area dedicated to rice cultivation increased by 124%, corresponding to a 215% increase in production (FAO, 2015). Upland rice varieties that are interspecific cultivars between *Oryza sativa* and *Oryza glaberrima* were developed by the Africa Rice Center under the name NEw RICe for Africa (NERICA; Jones et al., 1997a,b). These varieties promoted rice cultivation in the region and are partly responsible for the recent expansion of rice into rain-fed areas (Rodenburg et al., 2006; Balasubramanian et al., 2007; Wopereis et al., 2008). These areas are sometimes infested by the root-parasitic weed *Striga hermonthica* (Delile) Benth. Rice is a genuine host for *S.* *hermonthica* and heavy infestations by the parasite result in severe yield losses. *Striga* infestation and damage are expected to be exacerbated by the predominant low soil fertility, irregular rains and low-input (subsistence farming) conditions (Babiker, 2007).

Available control measures against *Striga*, including cultural, chemical, and biological methods, are either too expensive or too knowledge-intensive for subsistence farmers (Babiker, 2007). Under the prevailing farming conditions in sub-Saharan Africa, resistant crop varieties have been proposed as the most cost-effective and easy to adopt or deploy as an integral component of an integrated *Striga* management strategy (Ejeta, 2007). Various responses of rice varieties to *Striga* have been identified, although the number of resistant varieties is still limited (Rodenburg et al., 2010). The slow pace of development and deployment of *Striga*-resistant varieties is, majorly, due to a paucity of resistance sources, the complex genetics of resistance and limited knowledge regarding the specific mechanisms associated with resistance phenotypes (Amusan et al., 2008). Understanding the different types of resistance phenotypes facilitates development of selection methods and strategies to improve crop resistance to the parasite.

Research on rice has revealed several types of resistance phenotypes that are expressed in different stages of the *Striga* life cycle. As obligate hemiparasites, *Striga* seeds germinate only when exposed to host root-derived stimulants (López-Ráez et al., 2009). Host plants that produce lower amounts or less effective types of germination stimulants prevent parasite attachment, i.e., they have pre-attachment resistance. In a study using 18 NERICA varieties and their parents, NERICA1 and CG14 were shown to produce fewer germination stimulants and exhibit less frequent *Striga* infections compared with the other varieties (Jamil et al., 2011). Furthermore, host plants with post-attachment resistance prevent parasite development. Cissoko et al. (2011) reported that NERICA1 and NERICA10 exhibited the highest post-attachment resistance to *S. hermonthica* and *Striga asiatica* (L.) Kuntze. Recently Rodenburg et al. (2015) using a set of 25 rice varieties reported consistency between field and laboratory data on resistance to *S. hermonthica* and *S. asiatica*. A lowland rice cultivar, Nipponbare, has been reported to exhibit post-attachment resistance associated with an incompatibility in the cortex, endodermis, and root stele (Gurney et al., 2006; Swarbrick et al., 2008). Yoshida and Shirasu (2009) conducted detailed anatomical studies and proposed that mechanisms occurring subsequent to establishment of vascular connections contributed to the resistance of Nipponbare to *Striga*.

Although there are several *Striga-*resistant rice varieties, as noted above, crop resistance against one *S. hermonthica* population may not always be effective against other populations (Huang et al., 2012). *S. hermonthica*, an obligate outcrosser, is renowned for its genetic diversity and virulence variability (Yoshida et al., 2010). Furthermore, development of a virulent population over time from a subset of *Striga* individuals within a seed bank may lead to beakdown of resistance (Huang et al., 2012; Rodenburg et al., 2015). Cissoko et al. (2011) reported that NERICA1 showed high resistance to *S. hermonthica* collected from maize (*Zea mays* L.) in Kenya and sorghum [*Sorghum bicolor* (L.) Moench] in Sudan and to *S. asiatica* from maize in the USA and rice (cultivar Supa) in Kenya. However, it was susceptible to *S. hermonthica* collected from a field previously planted with NERICA1. In addition to differences in *Striga* populations, growth conditions such as *Striga* seed bank size, soil moisture, fertility, and temperature may influence apparent resistance in rice varieties (Kim, 1996)

Sudan is attempting to boost its domestic rice production and the area under the crop increased from 2,100 ha in 1984 to 7,562 ha in 2013 with a corresponding increase in production from 2,000 t to 25,000 t (FAO, 2015). Several NERICA varieties were introduced to Sudan and evaluated for growth and yield, but not for resistance to *Striga*, in the country (Somado and Guei, 2008). *Striga*, currently pre-dominantly on sorghum and millet, is expected to be a major production constraint to upland rice in Sudan as well. Expanding upland rice production without due attention to the *Striga* problem is fraught with an element of risk. Upland rice varieties which combine resistance to resident *Striga* populations and adaptability to Sudanese rice upland growing environments is therefore urgently needed.

The present investigation was therefore undertaken to (1) identify *Striga*-resistant upland rice varieties in a series of laboratory, pot and semi-controlled open air experiments using a resident *Striga* population and (2) determine, broadly, resistance phenotype(s) of the selected varieties. In Sudan, two distinct *S. hermonthica* strains, populations from sorghum fields and from millet fields, have been reported (Ali et al., 2009). We used a population from a sorghum field in this study, because millet cultivation area is often too dry for rice cultivation.

## Materials and Methods

### Plant Materials

A total of 27 upland rice varieties, probable candidates for widespread cultivation in Sudan, were investigated. Three rice varieties, Umgar (YUNLU 30 originating from China), Kosti1 and Kosti2, released for commercial production in Sudan (Nyachae, 2011), were obtained from the ARC, Sudan. Eighteen upland NERICA varieties and their parents, CG14, WAB56-50, WAB56-104, and WAB181-18, were supplied by the Africa Rice Center, Benin. Two lowland varieties, Nipponbare (*Striga*-resistant) and Kasalath (*Striga*-susceptible or -tolerant; Gurney et al., 2006; Swarbrick et al., 2009; Yoshida and Shirasu, 2009; Yoder and Scholes, 2010), were used as controls. Nipponbare and Kasalath were obtained from the GenBank of the National Institute of Agrobiological Science, Japan. A sorghum variety, Abu70, a high *Striga* germination stimulant producer and a positive control in evaluation of germination-inducing activity of root exudates, was provided by the ARC. *S. hermonthica* seeds were collected from a sorghum field near Wad Madani, Sudan, where upland rice cultivation is being conducted (Mustafa and Elsheikh, 2007).

### Rhizotron Experiment

Two rhizotron experiments were carried out under laboratory conditions as described by Hiraoka and Sugimoto (2008). The first experiment was conducted to evaluate post-attachment resistance of 27 rice varieties. The second was conducted to evaluate the repeatability and reliability of rhizotron technique using a subset of the varieties comprising NERICA5, Umgar, Nipponbare, Kasalath, and NERICA4. Seeds of each variety, sown on a moist filter paper and placed in a Petri dish, were incubated in the dark at 30°C for 4 days. Each seedling was transferred to a 10-mL test tube and grown hydroponically in 40% Long Ashton solution for 6 days. The seedlings were maintained in a growth chamber at 30°C with a 12-h photoperiod using fluorescent lights (photosynthetic active radiation of 220 μmol m^-2^ s^-1^). A 10-days-old seedling of each variety (with five replicates) was transferred to a rhizotron comprising a 15-cm Petri dish filled with rock wool overlaid by glass fiber filter paper watered with 40% Long Ashton solution. The seedlings were grown in the same growth chamber for additional 10 days. *Striga* seeds were surface sterilized by immersion in 0.75% (w/v) NaClO containing a few drops of Tween 20 and then subjected sonication for 3 min in an ultrasonic cleaner. After rinsing with distilled water and drying in a laminar hood, seeds were pre-treated (conditioned) for 12 days on 8-mm glass fiber filter paper disk (approximately 50 seeds each) placed on distilled water-saturated filter paper. Conditioned *Striga* seeds were treated, in separate Petri-dishes, with GR24 at 0.34 μM and incubated in the dark at 30°C for 1 day prior to inoculation. Roots of each 20-days-old rice seedling, in a rhizotron, were inoculated with 30 pre-germinated *Striga* seeds and subsequently incubated in the same growth chamber.

**FIGURE 1:**
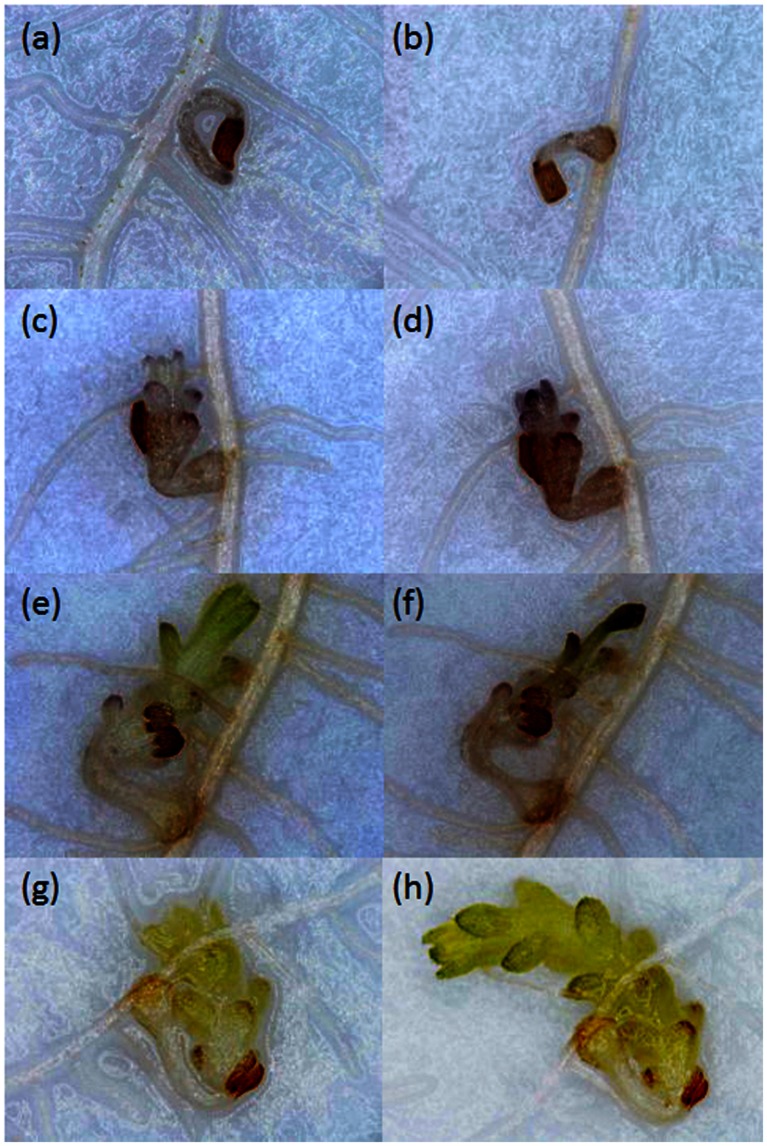
**Growth of *Striga hermonthica* seedlings inoculated onto rice roots in a rhizotron under 100× magnification.** **(a)** A seedling that failed to attach to the host root at 14 DAI. **(b)** A seedling that attached to the root but showed no shoot emergence at 21 DAI, or no shoot elongation from **(c)** 14 DAI to **(d)** 21 DAI. A seedling that **(e)** reached the four or more leaf pairs stage before 14 DAI and then **(f)** died before 21 DAI. A seedling that parasitized successfully with steady healthy shoot growth from **(g)** 14 DAI to **(h)** 21 DAI.

Growth of *Striga* seedlings was observed at 14 and 21 DAI using a stereomicroscope. Growth progression of *Striga* was classified into four developmental stages: stage I, failure to attach to the root due to lack of haustorium formation (**Figure 1a**); stage II, successful attachment but no shoot elongation (**Figures 1b–d**); stage III, shoot necrosis after reaching the four or more leaf pairs stage (**Figures 1e,f**); stage IV, successful parasitism with healthy shoots (**Figures 1g,h**).

### Pot Evaluation

Three pot experiments were carried out in the open air at the Sudan University of Science and Technology, Khartoum North (15°39′N lat, 32°31′E long) to compare resistance and performance of selected rice varieties under the same *Striga* seed bank size. Three rice varieties Kosti1, Kosti2, and Umgar were used for the first experiment. For the second experiment, six rice varieties were used. NERICA5, NERICA13, and Umgar were selected as potential candidates showing post-attachment resistance, based on the results of the rhizotron evaluation. NERICA18 was selected as a susceptible variety and NERICA4 was selected as it is widely used in several sub-Saharan African countries (Somado et al., 2008; Wopereis et al., 2008). The *Striga-*resistant variety Nipponbare, albeit a lowland type, was included as a resistant control. For the third experiment, NERICA4, NERICA5, NERICA13, Umgar, Kosti1, and Kosti2 were used. Nipponbare was not included because of lack of adaptability to upland rice growing environment confirmed in the second pot experiment and the semi-controlled open air experiment mentioned below here.

Plastic pots, 30-cm internal diameter and 30-cm height, perforated at the bottom were, each, filled with 9 kg of soil (vertisol, pH 8.31, EC 0.14 mS cm^-1^, total N 0.48 g kg^-1^, Olsen-P 8.15 mg kg^-1^, organic-C 8.9 g kg^-1^). Soil was collected from a *Striga* free plot within the college experimental farm. *Striga* seeds (1, 2, 4, 8, and 16 mg for the first experiment, 16 mg for the second and 16, 32, and 48 mg for the third) were mixed into the top 5 cm of soil in each pot. *Striga*-infested and *Striga*-free controls were included, as relevant, for comparison. Treatments were organized in a randomized complete block design with three replicates in the first experiments and six replicates in the second and third experiments. One day prior to sowing, 100 g of compost was mixed with the soil in each pot. On the day of sowing, 0.92 g of N as urea and 0.17 g of P_2_O_5_ as single superphosphate were added to each pot. Five rice seeds were sown in each pot on 30 June 2010, 5 September 2012, and 1 July 2013 for the first, second and third experiments, respectively. The seedlings were thinned to two per pot 14 DAS. Weather data were not recorded in the first experiment. During the experimental period, daily mean air temperature ranged between 18.1 and 34.0°C for the second experiment and between 24.4 and 36.4 for the third experiment. Total precipitation was 33.4 mm for the second experiment and 111.2 mm for the third and the average daily solar radiation was 16.5 and 18.6 MJ m^-2^ d^-1^, respectively. Rice was grown aerobically. Prior to heading, all pots were irrigated at 2-days intervals. After heading, plants were irrigated daily. Panicles were covered by paper bags 2 weeks after heading to prevent bird damage.

Emergent *Striga* plants per pot were counted weekly from 49 to 119 DAS for the first experiment, at 1140, 1453, 1907, 2112, 2305, 2493, 2689, 2875, 3023, and 3178 GDDs for the second experiment and at 1111, 1286, 1485, 1696, 1908, 2121, 2339, 2530, 3013, and 3203 GDD for the third. Rice was harvested by cutting the above-ground parts at ground level for the second and third experiments and no rice sampling was conducted in the first experiment. NERICA5 was harvested at 117 DAS for the second experiment and 98 DAS for the third and the other varieties were harvested at 125 DAS and 118 DAS, respectively. The rice above-ground parts, severed into panicles and vegetative parts, were oven dried at 80°C for 3 days and weighed. Panicle DW was used as an index for grain yield as described in studies of responses of sorghum varieties to *S. hermonthica* (Lendzemo and Kuyper, 2001; Van Ast and Bastiaans, 2006) and for rice in several studies (Kato et al., 2007; Ohe et al., 2010; Uga et al., 2011; Niones et al., 2012). Total above-ground parts DW was used as an index for rice growth, because it is an important factor for improving grain yield of rice (Fageria, 2007).

### Semi-Controlled Open Air Experiment

An experiment was conducted at the college farm to validate the results of the pot experiments under conditions that were relatively similar to farmer’s fields. During the experimental period (16 July 2012 to 14 November 2012) daily mean air temperature ranged between 26.1 and 34.8°C, relative humidity was 52.8% on average hourly measurements from sowing to 60 DAS. Low relative humidity, 37.5% on average hourly measurement, was predominant from 61 DAS onward. Total precipitation was 95.8 mm and the average daily solar radiation was 17.8 MJ m^-2^ d^-1^.

The same six rice varieties used in the second pot experiment were grown in adjacent *Striga*-free and *Striga*-infested plots. The *Striga*-infested plot was established artificially in the *Striga*-free area in 2011 using the same parasite population used in the previous experiments. The plots were used for sorghum cultivation in 2011 and before. *Striga* infestation in the infested plots was further augmented with 1 mg of the parasite seeds placed in each rice-planting hole. Treatments were arranged in a split-plot design. The *Striga* treatment was the main plot. The main plot was subsequently divided into four blocks. Rice planting and husbandry practices were as recommended by ARC, Sudan for upland rice production under supplementary irrigation. Each subplot was surrounded by ridges and rice was grown aerobically using flush irrigation. The crop was irrigated at 3- to 4-days intervals. The rice varieties were randomly allocated to the subplots (0.75 m × 3.75 m) within each block. Rice seeds were sown in holes (five per hole) with a spacing of 25 cm × 25 cm. P_2_O_5_, as single superphosphate (110 kg P_2_O_5_ ha^-1^) was applied at sowing. Nitrogen, as urea (165 kg N ha^-1^), was applied as split equal doses at 14, 35, and 49 DAS. Weeds other than *Striga* were manually removed. The field was covered with a nylon net to evade bird damage.

Emergent *Striga* plants around 10 fixed rice hills in each subplot were counted at 926, 1357, 1827, 2436, 2846, and 3333 GDD. Rice above-ground parts from the same 10 hills were harvested by cutting at ground level. NERICA5, NERICA13, NERICA18, and Nipponbare were harvested at 107 DAS. NERICA4 and Umgar were harvested at 111 and 121 DAS, respectively. The rice above-ground parts were severed into panicles and vegetative parts, oven dried at 80°C for 3 days, and weighed.

### Germination-Inducing Activity of Root Exudates

Seeds of the six rice varieties, used in the second pot and semi-controlled open air experiments, and those of a sorghum (cv Abu70), were surface sterilized by immersion in 1% (w/v) NaClO containing a few drops of Tween 20 and thoroughly rinsed with distilled water. The seeds sown on moist filter paper in Petri dishes were incubated in the dark at 30°C for 3–4 days for rice and 2 days for sorghum. The seedlings were transplanted each, to a 50-mL plastic tube wrapped in aluminum foil to exclude light. Plants were grown hydroponically in 40% Long Ashton solution in a growth chamber at 30°C with a 12-h photoperiod using fluorescent lights (photosynthetic active radiation of 220 μmol m^-2^ s^-1^) throughout growth duration after the transplanting except during root exudates samplings. The root exudate was sampled at 7, 14, 21, 28, and 35 DAS. One day before each sampling date, the roots were thoroughly rinsed with tap water and the nutrient solution in the plastic tubes was replaced with tap water. After 1 day incubation and prior to extraction, water loss was replenished in each tube and 100-μL of the aqueous solution containing root exudates was sampled, hence forth referred to as original exudates. The remaining aqueous solution in each tube was extracted with ethyl acetate (3 × 10 mL). The respective ethyl acetate extracts, pooled, dried over anhydrous sodium sulfate, were evaporated *in vacuo* at 40°C to dryness. The residues, were dissolved, each, in 1 mL of ethyl acetate. The original and concentrated root exudates were stored at -20°C, for at most 3 days, until completion of evaporating all samples at each sampling time. The germination bioassay followed the protocol described by Motonami et al. (2013) using *Striga* seeds that had been conditioned under sterilized conditions for 12 days on 8 mm diameter glass fiber disks (approximately 50 seeds per disk). Disks containing conditioned *Striga* seeds, dapped on a filter paper to remove excess water, were treated with an aliquot (20-μL each) of the original root exudates. Aliquots 20, 10, and 5-μL each, equivalent to 50, 25, and 10-time concentrated root exudates, respectively) of the concentrated extract were applied to a new 8-mm glass fiber disk. The disks were allowed to dry at room temperature for 2 h. Each treated disk was overlaid by a disk containing conditioned *Striga* seeds and moistened with distilled water (40 μL). The disks were incubated in the dark at 30°C for 1 day and examined for germination using a stereomicroscope. The synthetic germination stimulant GR24 was used at 0.34 μM as a positive control and tap water and distilled water were used as a negative controls. Three plants per variety were used and the root exudate from each plant was tested in five technical replicates (five disks).

### Statistical Analysis

The statistical package R (Version 3.0.2, R Core Team, 2015) was used for comparison of treatment means. In the rhizotron evaluation, the number of *Striga* seedlings in each growth stage was examined by Tukey’s HSD test with rice variety as a factor. In the pot and semi-controlled open air experiments, the number of emergent *Striga* plants and DW of rice varieties were analyzed by Student’s *t*-test or Tukey’s HSD test. The percentage values in germination tests were transformed to arcsine, compared by Tukey’s HSD test and back-transformed (Kgosi et al., 2012).

## Results

### Rhizotron Evaluation of Post-Attachment Resistance

**FIGURE 2:**
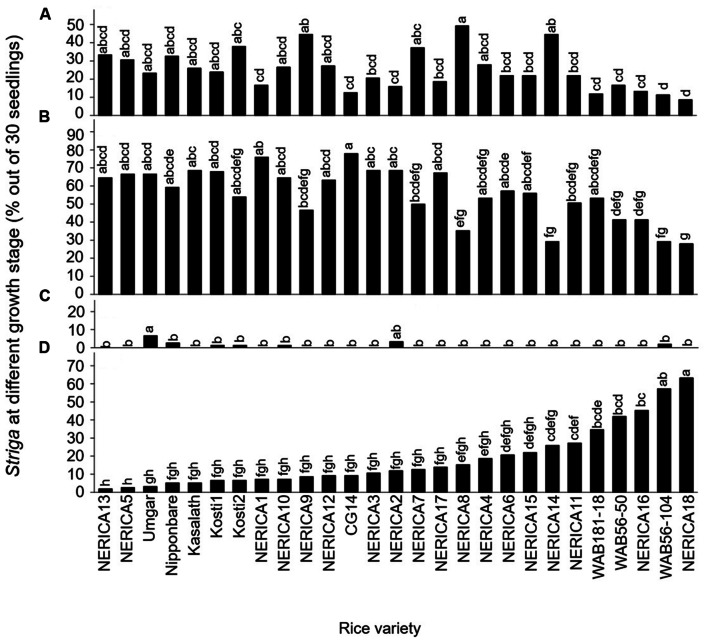
**Percentage out of 30 seedlings of *S. hermonthica* at stage **(A)** I, failure to attach to the root due to a lack of haustorium formation (**Figure 1a**); **(B)** II, successful attachment but no shoot elongation (**Figures 1b–d**); **(C)** III, shoot necrosis after reaching the four or more leaf pairs stage (**Figures 1e,f**); and **(D)** IV, successful parasitism with healthy shoots (**Figures 1g,h**) on the roots of 27 rice varieties in the first rhizotron experiment at 21 DAI.** Means followed by a common letter are not significantly different by Tukey’s HSD test in each growth stage (*p* < 0.05).

Developmental arrest of the parasite at stage I, failure in attachment, was common among all varieties (**Figure 2A**). Of the seedling inoculums, failure to attach to the host roots was less than 20, 20–30, 30–40 and more than 40% in 9, 10, 5, and 3 varieties, respectively. The developmental arrest at this stage was highest in NERICA8 (49.3%) and lowest in NERICA18 (8.7%).

Developmental arrest of the parasite at stage II, successful attachment but no shoot elongation, was common among all varieties (**Figure 2B**). Proportions of *Striga* seedling showing arrested development at this stage were less than 30, 30–40, 40–50, 50–60, and more than 60% in 3, 1, 3, 8, and 12 varieties, respectively. Successful attachment but no shoot elongation of *Striga* was highest in NERICA1 and CG14 (76.0–78.0%) and lowest in NERICA18 (28%; **Figure 2B**). Other than NERICA1 and CG14, there were 10 varieties, including NERICA13, NERICA5, and Umgar, in which more than 60% of the inoculated parasites showed arrested development at stage II (successful attachment but no shoot elongation).

The developmental arrest of *Striga* at stage III, necrosis of the parasite shoot, was observed in seven varieties (**Figure 2C**) and was highest in Umgar (6.7%). In this variety, 90% of the inoculated parasites underwent developmental arrest at stage I (failure in attachment) or stage II (successful attachment but no shoot elongation). These findings demonstrate that three *Striga* seedlings (10% of 30 seedlings) developed elongated shoots, but two of them (6.7% of 30 seedlings) died on Umgar. In other words, two-third of *Striga* seedlings that developed elongated shoots died on the variety.

The *Striga* seedlings at stage IV, classified as successful parasitism with healthy shoots, varied widely among varieties and represented 2–63.3% of the original inoculums (**Figure 2D**). Of the inoculated seedlings less than 10, 10–20, 20–30, 40–50, and more than 50% were maintained up to successful parasitism by 12, 6, 5, 2, and 2 varieties, respectively. NERICA18 retained a considerable proportion (63.3%) of the inoculated seedlings. In contrast, Nipponbare supported only 5.3% of the inoculated seedlings. Three varieties, NERICA13, NERICA5, and Umgar retained less than 5% of the inoculated seedlings up to stage IV (successful parasitism).

**FIGURE 3:**
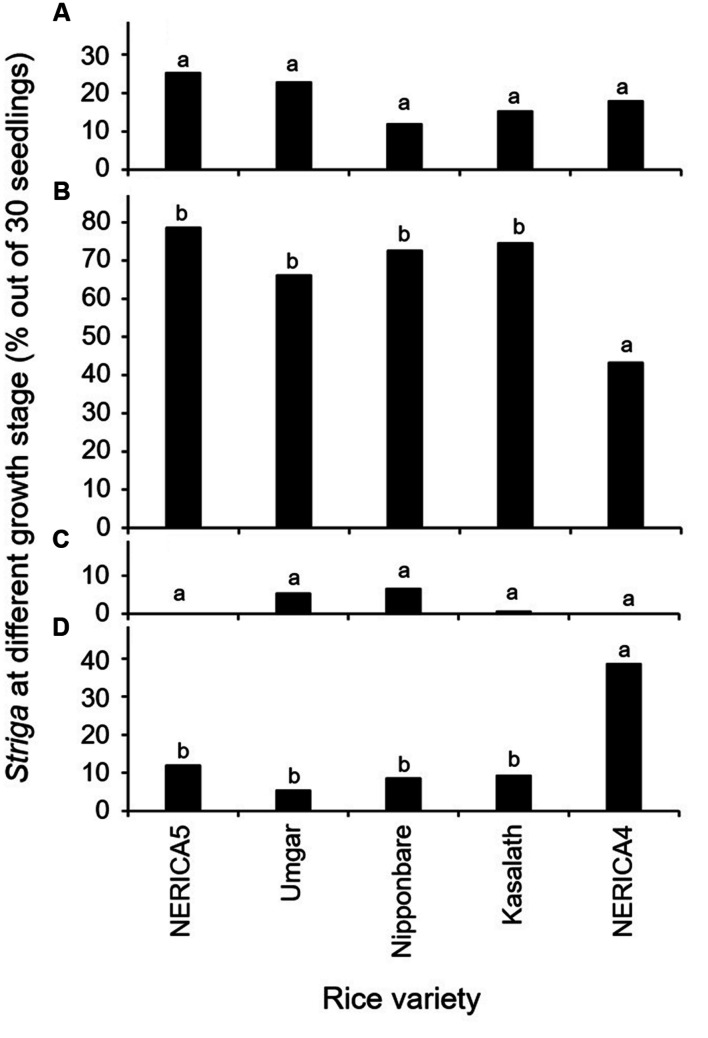
**Percentage out of 30 seedlings of *S. hermonthica* at stage **(A)** I, failure to attach to the root due to a lack of haustorium formation (**Figure 1a**); **(B)** II, successful attachment but no shoot elongation (**Figures 1b–d**); **(C)** III, shoot necrosis after reaching the four or more leaf pairs stage (**Figures 1e,f**); and **(D)** IV, successful parasitism with healthy shoots (**Figures 1g,h**) on the roots of five rice varieties in the second rhizotron experiment at 21 DAI.** Means followed by a common letter are not significantly different by Tukey’s HSD test in each growth stage (*p* < 0.05).

The results in the second rhizotron evaluation with a subset of the varieties (**Figure 3**) were similar to those in the first rhizotron evaluation (**Figure 2**). Developmental arrest of the parasite at stage I, failure in attachment, was much lower than that at Stage II, successful attachment but no shoot elongation in both evaluation (**Figures 2A,B and 3A,B**). Developmental arrest at stage III, necrosis of the parasite shoot, was observed repeatedly in Umgar and Nipponbare (**Figures 2C** and **3C**). *Striga* seedlings at stage IV, successful parasitism, on NERICA5, Umgar, Nipponbare, and Kasalath (2.7, 3.3, 5.3, and 5.3% in the first evaluation and 12.0, 5.4, 8.7, and 9.3% in the second evaluation, respectively) was much lower than on NERICA4 (18.7 and 38.7% in the first and second evaluation, respectively), albeit significant differences between the former four varieties and NERICA4 was observed only in the second evaluation (**Figures 2D** and **3D**).

### Pot and Semi-Controlled Open Air Evaluations of Selected Rice Varieties

**FIGURE 4:**
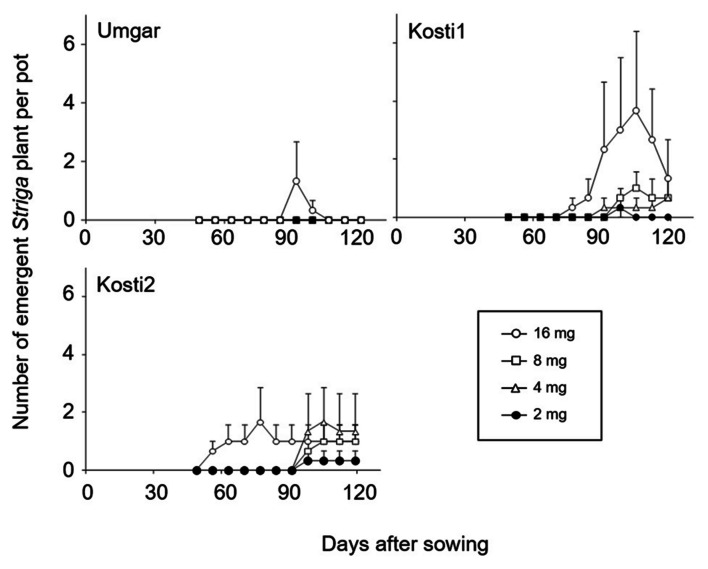
**Number of emergent *S. hermonthica* plants per pots planted with rice.** Umgar, Kosti1, and Kosti2 were used in the first pot experiment. The *Striga* seeds (2, 4, 8, and 16 mg) were mixed into top 5 cm of soil in each pot. Bars indicate standard errors of each mean value of three replications.

In the first pot experiments, Kosti1 supported 0, 0, 0.3, 0.7, 1.0, and 3.7 emergent *Striga* plants per pot at seed bank size of 0, 1, 2, 4, 8, and 16 mg *Striga* (**Figure 4**). The corresponding numbers for Kosti2 were 0, 0, 0.3, 1.7, 1.0, and 1.7, respectively. Umgar supported no emergent *Striga* plants at seed bank size of 0–8 mg. It supported 1.3 emergent *Striga* plants per pot at a seed bank size of 16 mg. However, the emergent *Striga* plants in Umgar pots died over a short period of time.

**FIGURE 5:**
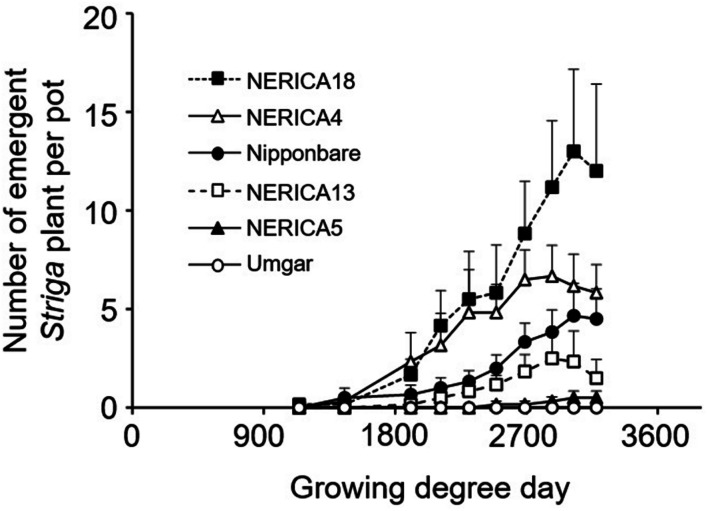
**Number of emergent *S. hermonthica* plants per pot planted with rice.** Umgar, NERICA5, NERICA13, Nipponbare, NERICA4, and NERICA18 were used in the second pot experiment. The *Striga* seeds (16 mg) were mixed into top 5 cm of soil in each pot. Bars indicate standard errors of each mean value of six replications.

In the second pot experiment, irrespective of rice variety, no *Striga* plants appeared in the *Striga*-free controls. Emergence of the parasite in the *Striga*-infested pots showed dependence on time and rice variety (**Figure 5**). Up to 1907 GDD, *Striga* emergence was lower than three plants per pot, irrespective of rice variety. However, the final parasite emergence reached 0, 0.5, 2.5, 4.7, 6.7, and 13.0 plants per pot for Umgar, NERICA5, NERICA13, Nipponbare, NERICA4, and NERICA18, respectively. There were significant differences (*p* < 0.05) between Umgar and NERICA18, between NERICA5 and NERICA18 and between NERICA13 and NERICA18. The difference between Nipponbare and NERICA18 was also significant (*p* < 0.10). *Striga* had no significant adverse effects (*p* > 0.10) on the total above-ground parts and panicle DW in Umgar, NERICA5, and NERICA13 (**Table 1**). However, significant reductions (*p* < 0.01 or 0.05) in total above-ground parts and panicle DW were observed in NERICA4, NERICA18, and Nipponbare (**Table 1**).

**Table 1:** Effects of *Striga hermonthica* on rice total above-ground parts and panicle DW in the second open-air pot experiment in 2012.

Variety	Total above-ground parts DW per pot (g)		Panicle DW per pot (g)	
		
	*Striga*-infested	*Striga*-free	*Striga*-infested	*Striga*-free
Umgar	39.8 ± 7.1	45.4 ± 5.0 NS	10.5 ± 1.2	8.8 ± 1.2 NS
NERICA5	18.8 ± 3.6	23.8 ± 4.8 NS	3.9 ± 1.1	4.6 ± 1.5 NS
NERICA13	23.0 ± 4.7	32.9 ± 7.5 NS	3.7 ± 1.5	3.5 ± 1.5 NS
NERICA4	10.3 ± 3.2	39.6 ± 9.3*	1.6 ± 0.7	6.4 ± 1.0**
NERICA18	15.1 ± 4.3	51.1 ± 3.9**	3.3 ± 0.8	7.5 ± 1.4*
Nipponbare	17.8 ± 4.5	31.5 ± 2.7*	2.9 ± 1.0	6.5 ± 1.2*


*Data are mean ± standard error of six replications. ^∗^ and ^∗∗^ indicate significant differences at *p* < 0.01 and 0.05, respectively, by Student t-test within a variety. NS indicates not significant (*p* > 0.10) within a variety.*

**FIGURE 6:**
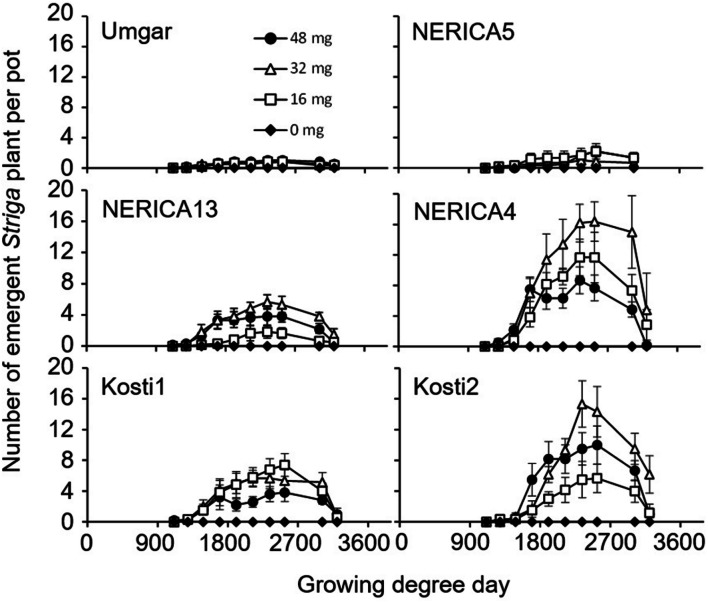
**Number of emergent *S. hermonthica* plants per pots planted with rice.** Umgar, NERICA5, NERICA13, NERICA4, Kosti1, and Kosti2 were used in the third pot experiment. The *Striga* seeds (0, 16, 32, and 48 mg) were mixed into top 5 cm of soil in each pot. Bars indicate standard errors of each mean value of six replications.

In the third pot experiment, irrespective of rice variety, no *Striga* plants appeared in the *Striga*-free controls. *Striga* emergence in the *Striga*-infested pots was lowest (2.2 plants per pot) on Umgar and NERICA5, irrespective of *Striga* seed bank size (**Figure 6**). NERICA13, at seed bank size of 16, 32, and 48 mg *Striga*, supported 1.8, 5.7, and 3.8 emergent *Striga* plants per pot, respectively. The corresponding numbers of emergent *Striga* plants were 11.7, 16.4, and 8.7 for NERICA4, 7.4, 5.7, and 3.8 for Kosti1 and 5.7, 15.3, and 10.0 for Kosti2, respectively. The differences between seed bank size of 16 and 32 mg was significant (*p* < 0.05) in NERICA13 and Kosti2. The deference between 32 and 48 mg was also significant for NERICA4 (*p* < 0.10). *Striga*, significantly (*p* < 0.05), reduced total above-ground parts DW of all varieties (**Table 2**) and the observed reductions were 17.2–36.8, 27.9–44.8, 74.5–84.9, 78.4–88.1, 84.6–86.1, and 71.8–75.9% for Umgar, NERICA5, NERICA13, NERICA4, Kosti1, and Kosti2, respectively.

**Table 2:** Effects of *S. hermonthica* seed density on rice total above-ground parts DW in the third open-air pot experiment in 2013.

	Total above-ground parts DW per pot (g)			
	
Variety	Amount or *Striga* seeds per pot (mg)			
	
	0	16	32	48
Umgar	48.2 ± 2.9a	39.9 ± 5.6ab	30.7 ± 3.9b	30.4 ± 3.8b
NERICA5	23.2 ± 2.1a	13.3 ± 3.1b	12.8 ± 1.4b	16.7 ± 1.9ab
NERICA13	32.3 ± 7.7a	8.2 ± 1.0b	4.8 ± 1.1b	4.9 ± 1.3b
NERICA4	29.1 ± 2.9a	3.7 ± 0.8b	6.3 ± 1.5b	3.5 ± 1.7b
Kosti1	22.3 ± 4.2a	3.1 ± 0.6b	3.4 ± 1.0b	3.2 ± 0.9b
Kosti2	22.5 ± 2.8a	5.9 ± 1.5b	6.4 ± 1.4b	5.4 ± 1.4b


*Data are mean ± standard error of six replications. Means within a variety followed by a common letter are not significantly different by Tukey’s HSD test (*p* < 0.05).*

**FIGURE 7:**
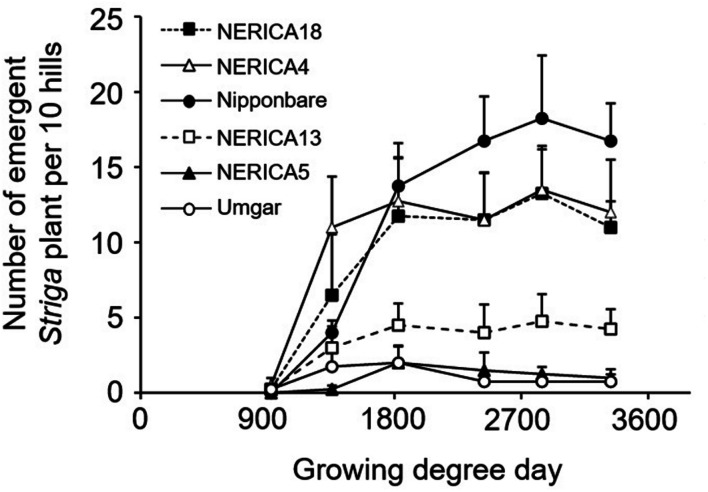
**Number of emergent *S. hermonthica* plants per 10 rice hills in an artificially infested field.** Umgar, NERICA5, NERICA13, Nipponbare, NERICA4, and NERICA18 were used in a semi-controlled open air experiment. Bars indicate standard errors of each mean value of four replications.

In the semi-controlled open air evaluation, irrespective of rice variety, no *Striga* plants appeared in the *Striga*-free controls. The number of emergent *Striga* plants per 10 rice hills in the *Striga*-infested field reached 2, 2, 4.8, 13.3, 13.5, and 18.3 for Umgar, NERICA5, NERICA13, NERICA18, NERICA4, and Nipponbare, respectively (**Figure 7**). There were significant differences (*p* < 0.05) between Umgar and Nipponbare, between NERICA5 and Nipponbare and between NERICA13 and Nipponbare. The differences between Umgar and NERICA18, between Umgar and NERICA4, between NERICA5 and NERICA18 and between NERICA5 and NERICA4 were also significant (*p* < 0.10). *Striga* had no significant adverse effects (*p* > 0.10) on total above-ground parts and panicle DW of Umgar, NERICA5, and NERICA13 (**Table 3**). However, the parasite reduced the total above-ground parts DW of NERICA4, NERICA18, and Nipponbare by 60.4% (*p* < 0.05), 43.1% (*p* < 0.05), and 45.3% (*p* < 0.10), respectively. Panicle DW was reduced by 69.5% (*p* < 0.10), 56.0% (*p* < 0.05), and 58.2% (*p* < 0.10) in NERICA4, NERICA18, and Nipponbare, respectively.

**Table 3:** Effects of *S. hermonthica* on rice total above-ground parts and panicle DW in a semi-controlled open air experiment in 2012.

Variety	Total above-ground parts DW (g m^-2^)		Panicle DW (g m^-2^)	
		
	*Striga*-infested	*Striga*-free	*Striga*-infested	*Striga*-free
Umgar	1010 ± 115	1000 ± 79 NS	293 ± 91	308 ± 41 NS
NERICA5	420 ± 65	378 ± 44 NS	128 ± 34	116 ± 25 NS
NERICA13	552 ± 82	543 ± 71 NS	143 ± 26	208 ± 55 NS
NERICA4	354 ± 140	894 ± 140*	92 ± 55	302 ± 80+
NERICA18	366 ± 70	643 ± 51*	84 ± 15	191 ± 32*
Nipponbare	196 ± 46	358 ± 62+	41 ± 11	98 ± 24+


*Data are mean ± standard error of four replications. ^∗^ and + indicate significant differences at *p* < 0.05 and 0.10, respectively, by Student t-test within a variety. NS indicates not significant (*p* > 0.10) within a variety.*

### Evaluation of Germination-Inducing Activity as Pre-Attachment Resistance

In all experiments tap water and distilled water induced negligible to little (0–5.4%) germination, while GR24 at 0.34 μM elicited over 90% germination (data not shown). Original root exudates of sorghum induced more than 80% germination. The 10, 25, and 50-time concentrated root exudates of sorghum induced 89.4, 96.4, and 95.1% germination, respectively. This confirms that the method used in our study could extract germination stimulants from hydroponic culture solutions.

**FIGURE 8:**
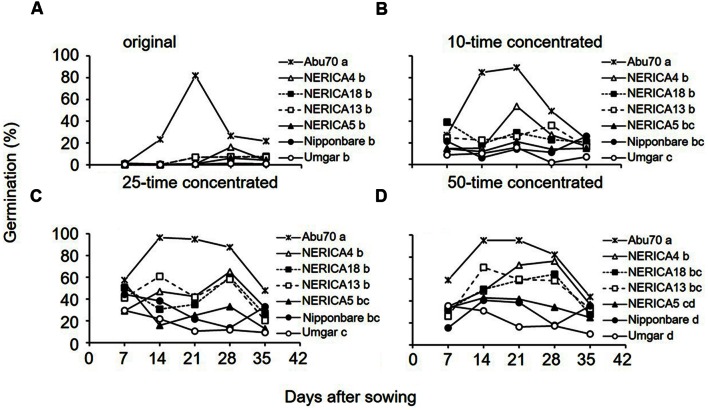
**Germination inducing activity of root exudates from Umgar, NERICA5, NERICA13, Nipponbare, NERICA4, and NERICA18 grown in a growth chamber.** Original **(A)**, 10-time concetrated **(B)**, 25-time concentrated **(C)** and 50-time concetrated **(D)** root exudates were used in the germintion test. The percentage values were arcsine transformed, compared by multiple mean-separation tests among rice and sorghum varieties and back-transformed. Different letters next to variety name indicate significant difference (*p* < 0.05) by Tukey’s HSD test pooled across sampling date.

The original root exudates from the rice varieties induced negligible to little (0.1–16.3%) germination (**Figure 8A**). Pooled across sampling dates, there was no significant (*p* > 0.05) difference among rice varieties. The 10 and 25-time concentrated root exudates from Umgar exhibited lower activity (1.9–29.6%) than those from NERICA4, NERICA13, and NERICA18 (14.7–65.0%) on all sampling dates (**Figures 8B,C**). Pooled across sampling dates, root exudates from Umgar exhibited significantly lower (*p* < 0.05) activities compared to those from NERICA4, NERICA13, and NERICA18 and there was no significant (*p* > 0.05) difference among Umgar, Nipponbare, and NERICA5. The 50-time concentrated root exudate from Umgar exhibited lower activity compared to those from the other rice varieties on all sampling dates, except at 7 DAS (**Figure 8D**). Pooled across sampling date, root exudates from Umgar and Nipponbare exhibited significantly lower (*p* < 0.05) activities compared to those from NERICA4, NERICA13, and NERICA18. Germination inducing activity from NERICA5 was intermediate among the six rice varieties.

## Discussion

Expanding upland rice cropping using irrigation water is one of options to boost domestic rice production in Sudan. Parasitic weeds thrive in production systems characterized by degraded soils with a lack of water control (Rodenburg et al., 2010). However, in the present study, obvious adverse effects from parasitism of a resident *Striga* population were observed on NERICA4 and NERICA18, probable candidates for widespread cultivation in Sudan, repeatedly in two pot experiments and a semi-controlled open air experiment with irrigation. These findings underpin the prediction that expanding upland rice production using supplementary irrigation in Sudan without due attention to the *Striga* problem is fraught with an element of risk. Identification of rice varieties with resistance to resident *Striga* populations and adaptability to the target environment is imperative to evade the risk.

The results of the rhizotron evaluation indicated that resistance to *S. hermonthica* is an outcome of several restrictions that block the parasite ingress and development. Failure of the parasite to attach to the host roots (stage I) due to lack of haustorium formation, is common to all varieties, however, inter-varietal differences were considerable. Inter-varietal differences in haustorium initiation is at variance with earlier views that haustorium initiation is not host discriminate and that host specificity is not associated with haustorium initiation, but rather with the ability of haustoria to functionally establish after invading the host (Riopel et al., 1990). However, recent research associated differential pre-attachment resistance to *S. asiatica* in wild sorghum with variability in haustorium-inducing activity (Rich et al., 2004; Bandaranayake and Yoder, 2013).

Developmental arrest of the parasite at stage II (successful attachment but no shoot elongation) contributed to the high post-attachment resistance in NERICA13, NERICA5, and Umgar. A similar resistance phenotype, namely successful attachment but no shoot elongation, has been reported in other rice varieties including Nipponbare, Koshihikari, CG14, NERICA1, and NERICA10 (Gurney et al., 2006; Swarbrick et al., 2008; Yoshida and Shirasu, 2009; Cissoko et al., 2011). However, the reasons for the developmental arrest are unclear and need to be ascertained. Although deposition of dense staining material(s) at the interface between NERICA10 and *Striga* has been observed (Cissoko et al., 2011), the mechanism was not always applicable to other cases. In addition, root endodermis structure did not explain the difference between a resistant variety (Nipponbare) and a susceptible variety (Kasalath; Gurney et al., 2006). Furthermore, hypersensitivity, which has been observed in sorghum (Mohamed et al., 2003), was not observed in this study.

The high post-attachment resistance of Umgar was partly attributable to the developmental arrest of *Striga* at stage III, namely shoot necrosis after reaching the four or more leaf pairs stage. The developmental arrest at this stage suggests inhibition following successful establishment of a vascular connection between the host and the parasite. Resistance following successful connection with host vascular system has been observed with several *Striga* and *Orobanche* species and is attributed to blockage of the host vessels and disruption of the flux of water, nutrients and carbohydrates from host to parasite (Arnaud et al., 1999; Amusan et al., 2008; Timko and Scholes, 2013). This resistance phenotype was not observed in NERICA13 and NERICA5, demonstrating that Umgar has additional resistance phenotype(s) which come(s) into play after establishment of connection with the host vascular system.

The results from the pot and semi-controlled open air experiments corroborated the strong *Striga*-resistance of NERICA5 as identified in the rhizotron experiment. NERICA5, irrespective of *Striga* seed bank size, sustained the lowest *Striga* emergence. The sharp decline in *Striga* emergence at the highest seed bank size (48 mg per pot) notable with the susceptible variety NERICA4 and Kosti2 could be due to intra-specific competition among the attached parasite seedlings and to severe inhibition of host growth. Interestingly, NERICA5 was reported as a variety with broad spectrum resistance to *Striga* species and ecotypes (Cissoko et al., 2011; Jamil et al., 2011; Rodenburg et al., 2015). The results of the current study, invariably, indicate the resistance of NERICA5 to another population of *S. hermonthica*.

The strong *Striga*-resistance of Umgar to a resident *Striga* population was also confirmed repeatedly in the rhizotron, the pot and the semi-controlled open air experiments undertaken in this study. Umgar is an upland variety released in Sudan for commercial production (Nyachae, 2011). Two other released rice varieties in Sudan, Kosti1 and Kosti2, did not exhibit *Striga*-resistance at the same level as Umgar. It must be taken into account that *Striga* seeds were mixed into the top 5 cm of soil in the pot experiments and the infested plot was further augmented with the parasite seeds placed in each rice-planting hole in the semi-controlled open air experiment. The possibility of having differential response of the rice varieties to *Striga* due to variations in root size and architecture and/or vertical distribution of the parasite seeds cannot be ruled out.

The lower germination inducing activity of root exudates from Umgar showed pre-attachment resistance. Although the germination inducing activity from NERICA5 did not show any significant difference (*p* > 0.05) to that from Umgar, the former was always higher than the latter, except with the test using original root exudates which would be too diluted to induce *Striga* germination. This finding suggest that Umgar has higher pre-attachment resistance than NERICA5. The mechanism of low induction of *Striga* seed germination by Umgar could be further investigated. The germination test using serial concentration ruled out the possibility of inhibition of *Striga* seed germination by high concentration of the stimulant. However, involvement of germination inhibitors cannot be ruled out as the solvent extraction may exclude hydrophilic inhibitors (Yoneyama et al., 2010).

NERICA13, as revealed by the rhizotron experiment, showed post-attachment resistance comparable to Umgar and NERICA5. However, it sustained higher *Striga* emergence in the pot and semi-controlled open air experiments. The differential performance of these varieties may be attributed to the relatively high germination inducing activity of root exudates of NERICA13 compared to the other varieties.

The results from rhizotron, pot and semi-controlled open air evaluations and germination test were at variance for Nipponbare. The rhizotron evaluation and germination test confirmed previous reports of a high *Striga* resistance (Gurney et al., 2006; Swarbrick et al., 2008; Yoshida and Shirasu, 2009). However, the results from pot and semi-controlled open air evaluations demonstrated a high *Striga* emergence. Notably, Nipponbare displayed poor growth and reached 50% heading very early at 44 DAS in the semi-controlled open air experiment (data not shown). The notable high *Striga* emergence could thus be due to the attendant stress and/or differences in ecotypes of *Striga* used by the previous workers (Gurney et al., 2006; Swarbrick et al., 2008; Yoshida and Shirasu, 2009) and the one used in this experiment. The poor growth and early flowering could be attributed, among other factors, to photoperiodic sensitivity (Matsubara et al., 2008). Nipponbare is a lowland variety and not adapted to aerobic conditions. The results also varied for Kasalath. Although, it was evaluated as *Striga*-susceptible or -tolerant in previous reports (Gurney et al., 2006; Swarbrick et al., 2009; Yoshida and Shirasu, 2009; Yoder and Scholes, 2010), it exhibited resistance comparable to Nipponbare repeatedly in the rhizotron experiments. The variance could be attributable to differences in *Striga* populations used in the previous studies and the present one. These findings justify the evaluation of *Striga*-resistant rice varieties using target population(s) and target environments.

## Conclusion

The relatively low germination inducing activity of root exudate together with the notable developmental arrest of the inoculated seedlings during stage II (successful attachment but no shoot elongation) and stage III (shoot necrosis) as revealed by the rhizotron experiments showed that Umgar possesses both pre- and post-attachment resistance. The high *Striga* resistance of Umgar was confirmed in pot and semi-controlled open air experiments conducted in Sudan. Its multiple resistance phenotypes to *Striga* would confer a wider ecological amplitude. NERICA5, which was reported as a variety with broad spectrum of resistance to *Striga* species and ecotypes, exhibited resistance against a population of *S. hermonthica* resident in Sudan. However, further studies including grain yield data under farmers’ conditions where *Striga* is pandemic is essential to evaluate environmental adaptability and usefulness of Umgar and NERICA5 in Sudan and to compare the results to other studies. Other rice varieties not included in pot and semi-controlled open air experiments in this study could express high *Striga* resistance, and therefore screening other rice varieties is highly recommended. Furthermore, spatial and temporal stability and durability of *Striga* resistance have to be further ascertained taking into account variability in environmental conditions and virulence between *S. hermonthica* ecotypes.

## Author Contributions

YS and HS conceived and designed the experiments with help of AB. HS performed the experiments with guidance of AM. HS, YS, and AB wrote the manuscript; all authors contributed to the discussion and approved the final manuscript.

## Conflict of Interest Statement

The authors declare that the research was conducted in the absence of any commercial or financial relationships that could be construed as a potential conflict of interest.

## Abbreviations

ARC: Agricultural Research Corporation
DAI: days after inoculation
DAS: days after sowing
DW: dry weight
GDD: growing degree days
